# Ultrasound in the diagnosis of a median neuropathy in the forearm: case report

**DOI:** 10.1186/1749-7221-2-23

**Published:** 2007-12-04

**Authors:** Stuart D Ginn, Michael S Cartwright, George D Chloros, Francis O Walker, Joon-Shik Yoon, Martin E Brown, Ethan R Wiesler

**Affiliations:** 1 Department of Orthopaedic Surgery, Wake Forest University School of Medicine, Winston-Salam, NC, USA; 2 Department of Neurology, Wake Forest University School of Medicine, Winston-Salem, NC, USA; 3 Department of Rehabilitation Medicine, Korea University College of Medicine, Seoul, South Korea

## Abstract

**Background:**

Electrodiagnostic studies are traditionally used in the diagnosis of focal neuropathies, however they lack anatomical information regarding the nerve and its surrounding structures. The purpose of this case is to show that high-resolution ultrasound used as an adjunct to electrodiagnostic studies may complement this lack of information and give insight to the cause.

**Case presentation:**

A 60-year-old male patient sustained a forearm traction injury resulting in progressive weakness and functional loss in the first three digits of the right hand. High-resolution ultrasound showed the presence of an enlarged nerve and a homogenous soft-tissue structure appearing to engulf the nerve. The contralateral side was normal. Surgery revealed fibrotic bands emanating from the flexor digitorum profundus muscle compressing the median nerve thus confirming the ultrasound findings.

**Conclusion:**

A diagnostically challenging case of median neuropathy in the forearm is presented in which high-resolution ultrasound was valuable in establishing an anatomic etiology and directing appropriate management.

## Background

The traditional diagnostic approach for focal neuropathies involves a detailed history and physical examination, augmented by electrodiagnostic studies (nerve conduction studies and electromyography). [1] While this approach is effective for localizing the site of pathology and determining the severity of the condition, it does have limitations. Electrodiagnostic studies are uninformative about structures surrounding the nerve and muscle, they do not allow visualization of intrinsic nerve or muscle abnormalities, and they are painful. High-resolution ultrasound (HRUS) is a non-invasive, painless, portable, and inexpensive modality that has become an attractive adjunct to electrodiagnostic studies in the evaluation of entrapment neuropathies. [2]

We present a diagnostically challenging case of median neuropathy in the forearm in which HRUS was used to direct appropriate management. This case illustrates that HRUS can be a useful complement to electrodiagnostic studies in the evaluation of focal neuropathies.

## Case presentation

A 60 year-old right-handed man with a history of degenerative cervical disc disease presented with complaints of right hand and forearm weakness that started 6 months earlier following an acute traction injury sustained while moving a large mattress. The mattress fell and pulled his right arm, and he immediately felt pain in his shoulder and elbow. Two hours after the injury he noticed weakness in the first three digits of his right hand.

One month later the weakness persisted, but it had not worsened. His primary care physician was initially concerned about cervical root trauma given his history of degenerative disc disease and the nature of the injury, but an MRI and CT myelogram of the cervical spine showed no changes compared to his previous cervical spine images. It was then assumed that he had a brachial plexus injury, and the plan was to follow his course clinically.

Over the next several months he developed progressive numbness over the palmar aspect of the first three digits, and progressive weakness in his hand and forearm. He also noted atrophy of the muscles in his volar forearm. Eight months after the initial injury he presented to our electromyography (EMG) laboratory. On examination he had profound weakness of the flexor pollicis longus and flexor digitorum profundus to the index and middle fingers, and mild weakness of the flexor digitorum superficialis, flexor carpi radialis, and abductor pollicis brevis. He also had decreased sensation over the palm in the distribution of the median nerve. Motor and sensory nerve conduction studies showed no response from the median nerve, and EMG localized the lesion as a focal neuropathy of the median nerve distal to the branch to the pronator teres muscle.

**Figure 1:**
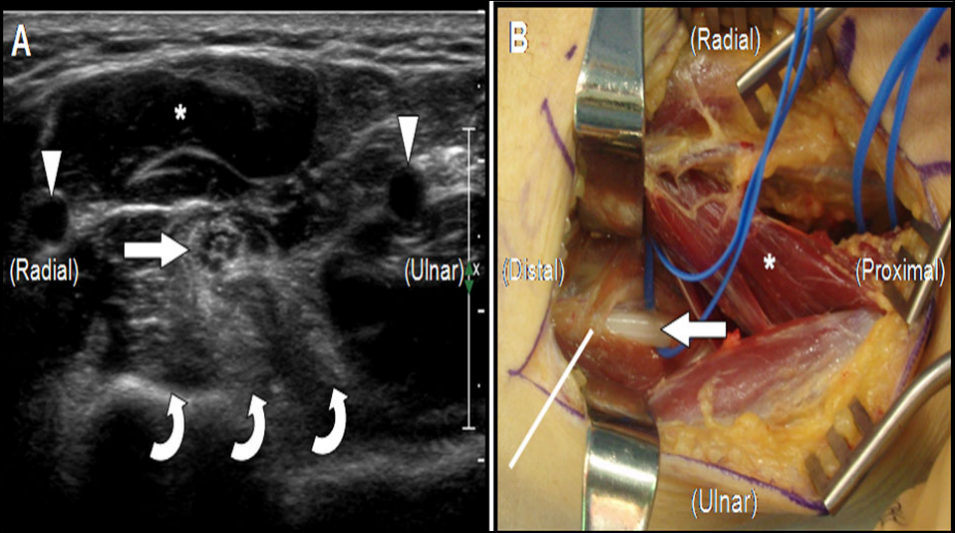
The cross-sectional ultrasound image (A) of the proximal forearm demonstrates the normal echo-texture of the median nerve (arrow). The hyperechoic and homogenous ground glass appearance of the flexor digitorum profundus muscle (curved arrows) is also shown. The intra-operative photo (B) depicts a fibrotic band (straight line) across the anterior aspect of the median nerve (arrow). Arrowheads = arteries, * = pronator teres muscle. The ultrasound image was obtained with a Philips iU22 scanner (Philips Medical Systems, Bothell, WA) with a 12 MHz linear array transducer.

HRUS using a Philips iU22 scanner (Philips Medical Systems, Bothell, WA) with a 12 MHz linear array transducer was performed to further explore this focal neuropathy. The median nerve was shown to be intact throughout the arm. At the presumed site of neuropathy the cross-sectional area of the nerve was enlarged, from 10.9 mm2 at the wrist to 17.2 mm2 at the site of maximal enlargement in the proximal forearm, but it maintained a normal echo-texture. The soft tissue deep to the median nerve at this site was hyperechoic and homogenous and appeared to engulf the nerve (Figure 1). Ultrasound of the corresponding level of the contralateral forearm demonstrated normal appearing muscle in clear contrast to the symptomatic arm.

Approximately one year had passed since the initial injury and based on the progressive weakness, new sensory findings, and ultrasonographic changes, median nerve exploration in the proximal forearm with planned neurolysis was pursued. A longitudinal incision was made in the anterior forearm just distal to the antecubital fossa. The median nerve was identified, surrounded by healthy pronator teres and flexor digitorum superficialis muscles. Initial intraoperative nerve conduction studies showed no response from the median nerve. Deep to the median nerve the flexor digitorum profundus to the index finger was found to be atrophic and fibrotic, and multiple rigid fibrous bands emanated from the muscle. Several of these bands crossed over and compressed the median nerve, both proximal and distal to the anterior interosseous nerve (Figure 1). These bands were released and intraoperative nerve conduction studies were repeated, again with no response from the median nerve.

Tendon transfers were performed to improve function. AIN reconstruction was foregone due to the low probability of functional improvement given the extensive fibrosis observed in the FDP muscle tissue. The viable flexor digitorum profundus to the ring finger was attached to the flexor digitorum profundus to the index finger with side-to-side tenodesis, and the flexor carpi radialis was transferred to the distal flexor pollicis longus through an incision at the wrist. The post-operative course was uncomplicated, and two months after the procedure the patient had improved hand function, consisting of slow, partial return of his sensory recovery, improved motor function and grip strength.

## Conclusion

The use of HRUS in peripheral nerve surgery is a relatively novel concept. To date, the majority of the studies using HRUS in peripheral nerves of the upper extremity have focused on the entrapment neuropathies of the median nerve at the wrist and of the ulnar nerve at the elbow. These studies have shown that HRUS is as a low-cost, non-invasive, painless adjunct to the nerve conduction studies in the diagnosis of these entities and have highlighted that in addition to nerve conduction studies, HRUS may further provide anatomic information that might help determine the cause. [3-6] Furthermore, recent studies have used HRUS to assess the morphologic changes of the median nerve after carpal tunnel syndrome release, [7] the presence of nerve transections, [8] and primary peripheral nerve repair. [9] In addition, a previous study showed ultrasound to be helpful in the pre-operative evaluation of nerve injuries. [10]

There are many potential causes of median nerve compression and injury in the forearm, including masses extrinsic or intrinsic to the nerve, trauma, anatomic anomalies, and entrapment. [11-14] HRUS can greatly improve diagnostic yield by identifying the specific anatomic etiologies responsible for the nerve pathology and was particularly useful in delineating the nature of median nerve involvement in this case. The median nerve was found to be intact throughout the forearm, which ruled out primary injury or transection of the nerve. Enlargement of the median nerve at the site of the neuropathy was consistent with compression-induced neuropathy, as is seen with entrapment at other sites, and this finding identified the specific site of neuropathy. [5,6] There were no ultrasonographic changes to suggest the presence of a neuroma. Finally, the abnormal appearance of the soft tissue deep to the median nerve in the anatomic location of the flexor digitorum profundus was consistent with an inflammatory or fibrotic process engulfing the median nerve, which prompted the decision to pursue surgical exploration and excision of the compressing tissue. The ultrasonographic findings were confirmed during surgical exploration of the forearm, where the abnormal soft tissue structure visualized by ultrasound corresponded to fibrous bands originating from the flexor digitorum profundus and entrapping the median nerve.

The mechanism of injury and the sequence of events that led to median neuropathy in this case are unclear, however, based on the history and ultrasound findings, we can make speculations. One possibility is that the initial traction injury damaged the anterior interosseous nerve, which resulted in the initial weakness without sensory changes. The absence of innervation to part of the flexor digitorum profundus caused this muscle to atrophy and fibrose, and some of the fibrotic tissue formed rigid bands that compressed the median nerve. The compression led to the development of a focal neuropathy, which was localized with ultrasound as an increase in median nerve cross-sectional area. Alternatively, the initial injury could have caused a tear of the flexor digitorum profundus muscle to the index finger, with the development of fibrotic bands compressing the median nerve during subsequent healing.

It has been shown that HRUS may be used as an adjunct to physical examination and electrodiagnostic findings in the diagnosis of nerve entrapment neuropathies in the absence of anatomic abnormalities. [3,5] This case demonstrates that it may be valuable in establishing an anatomic etiology and directing appropriate management in a diagnostically challenging case of median neuropathy in the forearm. In addition, ultrasound is non-invasive, inexpensive, and effective as a pre-operative planning tool for the surgical treatment of focal neuropathies.

## Competing interests

Dr. Cartwright has a Clinical Research Training Grant from the Muscular Dystrophy Association to study neuromuscular ultrasound; however, this organization does not have a financial interest or conflict with the content of the manuscript. The other authors declare that they have no competing interests.

## Authors' contributions

SG performed all pertinent research and drafted the manuscript. MC, FW, and EW conceived the case report, performed evaluations and treatments for the patient, and helped to edit the manuscript. EW performed the patient's surgery. JY and GC helped to conceive of the study and participated in the editing process. MB performed the electrodiagnostic studies in the neurology clinic. All authors read and approved the final manuscript.
